# The Association Between Dietary Inflammatory Index and Sex Hormones Among Postmenopausal Women in the US

**DOI:** 10.3389/fendo.2021.771565

**Published:** 2021-12-21

**Authors:** Wen-Yu Chen, Yan-Peng Fu, Wen Zhong, Min Zhou

**Affiliations:** ^1^ Cardiovascular Medicine Department, Nanchang University Second Affiliated Hospital, Nanchang, China; ^2^ Second Clinical Medical College of Nanchang University, Nanchang, China; ^3^ Endocrinology Department, Nanchang University Second Affiliated Hospital, Nanchang, China

**Keywords:** dietary inflammatory index, diet, inflammation, androgens, estrogens, hormones

## Abstract

**Aims:**

Diet has been found to have an important effect on sex hormones. The effect of diet-induced inflammation on sex hormones has not been studied in detail among women. Therefore, we aimed to investigate the association between energy-adjusted dietary inflammatory index (E-DII) and sex hormones among postmenopausal women.

**Methods:**

This study used data from the National Health and Nutrition Examination Survey (NHANES) 2013–2016 waves. A total of 1183 postmenopausal women who provided information on two 24-hour dietary intake recalls, sex hormones including total testosterone (TT), estradiol (E2), TT/E2, sex hormone-binding globulin (SHBG), free estradiol (FE2) and free testosterone (FT), as well as selected covariates were included. Linear regression and restricted cubic spline evaluated the association between E-DII and sex hormones. Effect modification by body mass index (BMI) and type of menopause was then examined in stratified analysis.

**Results:**

After adjusting for covariates, linear regression showed that E-DII was positively associated with TT (*P*=0.035), FT (*P*=0.026) and TT/E2 (*P*=0.065). TT (*P*-nonlinear = 0.037) and TT/E2 (*P*-nonlinear = 0.035) had significant nonlinear association with E-DII. E2 (*P*-nonlinear = 0.046) and FE2 (*P*-nonlinear = 0.027) depicted a nonlinear U-shaped significant association with E-DII, the two inflection points were found at the E-DII score of -0.22 and 0.07, respectively, the associations in natural menopausal women were more pronounced.

**Conclusions:**

Our study indicates that several indicators of androgen and estrogen were associated with E-DII in postmenopausal women. Further research is needed to understand the underlying mechanisms.

## Introduction

It is well known that diet has an important effect on sex hormone-related metabolism and reproductive function (1–3). Dietary components have been studied that could modulate sex hormones, like total testosterone (TT) and estradiol (E2) (2, 4, 5). More than one dietary component was taken at each meal, the association between combined effect of various foods and sex hormones is worth exploring than for individual components (6, 7). A series of dietary index was created to study the overall effect of diet on individual outcome from all sides (8, 9). Rapid advances in recent years have improved our understanding of the role of diet-induced inflammation (10, 11). The dietary inflammatory index (DII) was developed as the inflammatory potential of the diet (12). Inflammation is an essential factor in the development and progression of many chronic diseases. An inflammatory state may be modified by diet (13). DII related with C-reactive protein (CRP), interleukin-6 (IL-6), tumor necrosis factor-α (TNFα), fibrinogen and white blood cell counts (14). The DII has been widely studied to provide new insight to the association between DII and various diseases, such as various cancer, cardiovascular diseases, and endocrine disruptions (15, 16). In parallel, some study has revealed that the suppression or promotion of inflammation was related to sex hormones (17). It could be inferred that the diet-induced inflammation may have impact on sex hormones. However, these studies on the association between the dietary inflammatory index (DII) and sex hormones are very limited. A few recent studies have begun to investigate the link between DII and sex hormones in men. A study of adult men (> 20 years old) in US found that higher DII score were associated with a higher risk of testosterone deficiency (18). Another study utilizing data from the NHANES found that DII score was negatively correlated with TT and E2 level in male adolescents (12-19 years old), while no correlation between them was observed in male children (6-11 years old) (19). In contrast, among male university students of 18-23 years old from Murcia Region (Southern Spain), no association between DII and sex hormones was observed (20). However, the association between sex hormones and DII among postmenopausal women has been not investigation so far.

Women after menopause are always accompanied by an increased risk of all-cause and cardiovascular disease death, perhaps attributing to the abrupt imbalance of sex hormones (21, 22). Among premenopausal and older women from InCHIANTI Study, inflammatory markers were found to be associated with lower concentrations of testosterone and E2, and higher concentrations of sex hormone-binding globulin (SHBG) (23). Moreover, the effects of pro- or anti- inflammatory dietary patterns for the incidence of sex hormone-related diseases in postmenopausal women are obvious (1, 2, 24). Therefore, the possible effect of DII on sex hormones in postmenopausal women may lead to an increased risk of sex hormone-related metabolic disorders or cancer in this vulnerable population. These findings lead us to evaluate the association between DII and sex hormones in postmenopausal women using data from the National Health and Nutrition Examination Survey (NHANES). Our study brings a new insight to explore underlying mechanistic pathways on sex hormone-related diseases induced by the inflammatory potential of diet in postmenopausal women.

## Materials and Methods

### Study Design and Population

NHANES is an ongoing cross-sectional survey of representative samples of the civilian non-institutionalized US population, conducted by the CDC’s National Center for Health Statistics (NCHS) (25). Study participants were sampled through a complex multistage probability sampling design. Two NHANES cycles (2013-2014 and 2015-2016) in this study included a total of 1183 US postmenopausal women with complete data on sex hormones (serum TT, E2, SHBG), two 24-h dietary recalls and covariates (listed below). Participants in this study who had total energy intakes outside the range of 500-5000 Kcal/day were excluded. The NHANES obtained approval from the National Center for Health Statistics Research Ethics Review Board and participants provided written consent.

### Exposure and Outcome Definitions

The energy-adjusted dietary inflammation index (E-DII) is regarded as an exposure variable. Consider that overall consumption of dietary energy is associated with the total inflammatory potential of the diet, E-DII was created (26). NHANES collected two 24-hour dietary recalls (24HRs) to accurately obtain dietary information. E-DII score was calculated based on a regionally representative world database which contains standard means and deviations for the 45 food parameters from 11 countries. 27 food parameters are available in NHANES, which included carbohydrates, protein, fat, alcohol, fiber, cholesterol, (saturated, monounsaturated, and polyunsaturated) fatty acids, omega-3 and omega-6 polyunsaturated fatty acids, niacin, vitamins (A, B1, B2, B6, B12, C, D, E), iron, magnesium, zinc, selenium, folic acid, beta carotene, and caffeine. The intake of each food parameter was adjusted by 1000 kcal, which requires using the energy-standardized version of the world database. The Z score was created for each food parameter by subtracting the adjusted global daily mean intake from the adjusted participant’s intake, and the value was divided by its standard deviation, next converted to a percentile and multiplied by 2 and subtracted from 1, then the percentile value was multiplied by the score of inflammatory effect of corresponding food parameter. The participants’ E-DII score was the sum of each E-DII score. Higher E-DII scores indicate more pro-inflammatory diets, and lower E-DII scores indicate more anti-inflammatory diets.

Sex hormones are regarded as outcome variables, which included TT, E2, SHBG, free testosterone (FT), free estradiol (FE2), TT/E2. Serum was separated from subsample blood obtained at MECs and stored at −70°C until the analytical laboratories in NHANES. TT, E2 and SHBG concentrations were quantified by competitive electrochemiluminescence immunoassays on a 2010 Elecsys autoanalyzer (Roche Diagnostics, Indianapolis, IN). The lowest detection limits of the assays and the coefficients of variation for quality control specimens were reported previously (27). NHANES files can be accessed for more details (25). TT/E2 were defined as the ratio of TT to E2, respectively. FT and FE2 are defined as the “bioavailable fraction”. Using equations based on the mass action law (28), FT was calculated from TT, albumin, and SHBG concentrations, FE2 was calculated from E2, albumin, and SHBG concentrations.

### Covariates

Covariates were considered as possible confounders based on relevant studies regarding sex hormones and diet (2, 3, 17). 12 covariates included in all adjusted analyses were: age, body mass index (BMI), waist circumference, parity, time since menopause, and energy intake were measured as continuous variables; race, education, marital status, type of menopause, time of blood sampling and smoking were assessed as categorical variables. Race was classified as Hispanic, non-Hispanic White, non-Hispanic Black, and other race. Education level was classified as less than high school, high school or equivalent, and more than high school. Marital status was classified as married or with partners, widowed or divorced, and unmarried. Smoking status was classified as never, former, and current smoker based on former literatures (29). Participants who had smoked less than 100 cigarettes in life were classified as never smokers; those who had smoked at least 100 cigarettes in life and now quitted smoking were classified as former smokers; those who had smoked at least 100 cigarettes in life and now continued smoking were classified as current smokers. Time of blood sampling (morning, afternoon, evening) was included considering those sex hormones have a strong circadian rhythm.

### Menopausal Status Definitions

**Figure 1:**
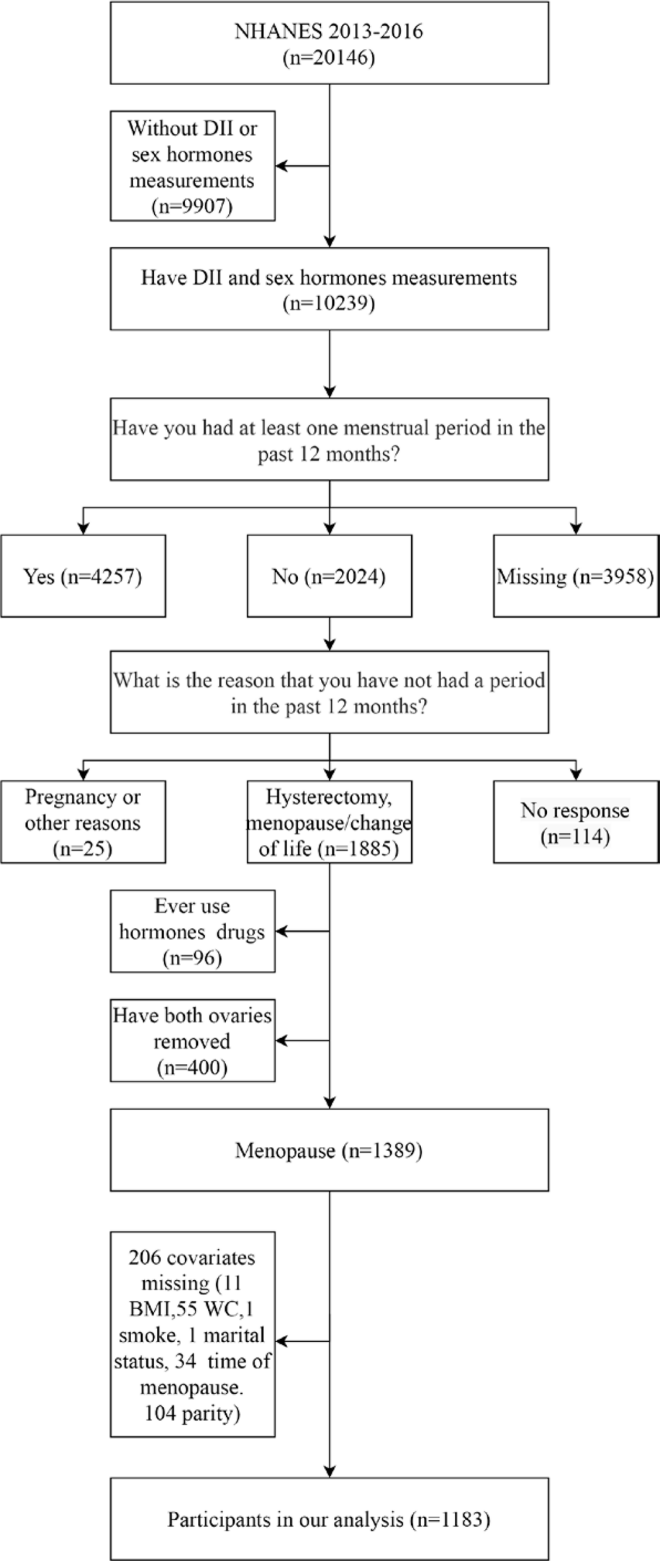
Flow chart of the selection of the study participants.

Menopausal status was defined based on a self-reported reproductive health questionnaire. We regarded women as postmenopausal who answered “no” to the question “Have you had at least one menstrual period in the past 12 months? and subsequently answered “hysterectomy” or “menopause/change of life” to the question “What is the reason that you have not had a period in the past 12 months?”. Then, 1885 postmenopausal women who had no available diet information or sex hormone measurements were excluded. Another 96 postmenopausal women were excluded due to a history of sex hormone medication use and 400 because of oophorectomy. An additional 206 participants with missing data on covariates were excluded: 104 missing parity, 11 missing BMI, 55 missing waistlines, 2 missing marital status, and 34 missing times since menopause. After missing data were excluded, 1183 postmenopausal women were available for our analysis (**Figure 1**).

### Statistical Analysis

All statistical analyses were computed with R version 3.4.3. We calculated weighted means (± weighted standard deviation) using the NHANES primary sampling unit, strata, and weights of diet subsamples for continuous variables and frequencies (weighted proportions) for categorical variables. Weighted chi-square test for categorical variables and weighted t-test for continuous variables were used to calculate for differences among the E-DII (tertiles) groups. Since The distributions of sex hormones were right-skewed, log transformation using the formula (ln) was applied to improve the normality in the linear regression analysis.

Unadjusted model and adjusted linear regression models were applied to investigate the linear association of the E-DII with TT, E2, SHBG, FT, FE2, and TT/E2. the E-DII (tertiles) was used as an ordinal variable for tests for trend. The restricted cubic spline model with three knots equally space within the range of the E-DII was applied to investigate the non-linear association. The Wald test was used to access non-linearity and P for trend. Multiple linear regressions with “rcs” function in R package “rms” were implemented. Three knots defined at the 10th, 50th, and 90th percentiles of E-DII scores were prespecified. Covariates as mentioned above were included in all adjusted analyses. An inflection point was determined as the one, at which a second derivative of a model turned zero. Akaike’s Information Criterion (AIC) was used to determine which model fits the data better (30). If AIC value of the restricted cubic spline model is smaller than the linear model, the former was regarded as the better-fitted model to describe the association of exposure and outcome variables.

Subgroup analysis was implemented to explore the effect modification by type of menopause (natural menopause, hysterectomy) and BMI (BMI<25kg/m2, BMI=25-29.9kg/m2, BMI≥30kg/m2). Concentrations of steroid hormones would be different in natural menopause and hysterectomy, and moreover, obesity would affect conversion of steroid hormones (17, 24). For these reasons, the above factors were selected as grouping variables. All regressions above were statistically weighted. 4-year sample weight is equal to two-year diet subsample weight divided by 2. A sensitivity analysis was performed to reanalyze dataset and test the stability of our main results using linear regression models and restricted cubic spline model without weighting. The statistical significance level was set at 0.05 (two-tailed).

## Results

### Population Characteristics

A total of 1183 postmenopausal women from the NHANES 2013-2016 were included. **Table 1** lists the characteristics of postmenopausal women overall and across the E‐DII tertiles. The E-DII score ranged from -5.04 (most anti-inflammatory) to 4.55 (most pro-inflammatory). They were on average 61.8 years old, approximately half (44.8%) of them were obese (BMI ≥ 30), and most of them (75.8%) underwent menopause naturally. Compared with those in the first tertile of E‐DII score, they in the third tertile were more likely to be younger, fatter, less educated. They also had more energy intake and were more likely to smoke (All *P <*0.05). TT concentration was significantly different in the different tertiles of E‐DII.

**Table 1:** Participant’s characteristics in NHANES 2013–2016 (N = 1183).

Characteristic	Total	Tertile 1	Tertile 2	Tertile 3	*P*
Age (yr), Mean (SD)	61.8 (10.3)	62.4 (9.9)	62.5 (10.7)	60.4 (10.1)	0.03
Race, n (%)					0.01
Hispanic	370 (12.7)	135 (14.1)	132 (13.3)	103 (10.6)	
Non-Hispanic White	482 (70.7)	140 (67.7)	167 (73.7)	175 (70.5)	
Non-Hispanic Black	227 (9.8)	64 (7.1)	72 (9.4)	91 (12.9)	
Other Race	104 (6.8)	56 (11.2)	23 (3.6)	25 (5.9)	
Education, n (%)					<0.001
Less than high school	302 (16.6)	105 (16.6)	113 (18.9)	84 (14.1)	
High school or equivalent	295 (24.3)	75 (15.0)	95 (20.7)	125 (37.3)	
More than high school	586 (59.2)	215 (68.3)	186 (60.4)	185 (48.6)	
Marital status, n (%)					0.002
Married or with partners	676 (64.9)	246 (73.8)	207 (59.5)	223 (61.9)	
Widowed or divorced	437 (32.2)	127 (24.2)	172 (39.2)	138 (33.0)	
Unmarried	70 (2.8)	22 (2.1)	15 (1.4)	33 (5.2)	
Smoke, n (%)					<0.001
Non-smoker	729 (60.0)	294 (73.5)	229 (54.6)	206 (52.1)	
Former smoker	269 (24.7)	75 (20.7)	111 (31.5)	83 (21.6)	
Current smoker	185 (15.3)	26 (5.8)	54 (13.8)	105 (26.4)	
BMI (kg/m^2^), Mean (SD)	30.1 (7.1)	28.4 (6.3)	30.4 (7.2)	31.4 (7.4)	0.009
Normal < 25	276 (26.2)	110 (35.8)	95 (25.0)	71 (17.7)	0.02
Overweight 25~29.9	347 (29.0)	125 (27.5)	105 (28.0)	117 (31.6)	
Obese ≥30	560 (44.8)	160 (36.7)	194 (47.0)	206 (50.7)	
Waist circumference (cm), Mean (SD)	100.4 (15.4)	96.4 (14.9)	101.4 (15.6)	103.4 (14.7)	0.003
Time since menopause (yr), Mean (SD)	15.0 (11.3)	14.3 (10.7)	16.0 (12.2)	14.7 (11.0)	0.45
Type of menopause, n (%)					0.02
Hysterectomy	288 (24.2)	86 (16.3)	83 (25.1)	119 (31.1)	
Natural menopause	895 (75.8)	309 (83.7)	311 (74.9)	275 (68.9)	
Parity, Mean (SD)	3.4 (1.9)	3.5 (1.8)	3.4 (1.8)	3.4 (2.0)	0.95
Time of blood sampling, n (%)					0.47
Morning	591 (45.8)	205 (47.6)	202 (46.2)	591 (43.8)	
Afternoon	449 (39.9)	145 (35.2)	145 (40.0)	159 (44.6)	
Evening	143 (14.1)	45 (17.2)	47 (13.8)	51 (11.6)	
Energy intake (kcal), Mean (SD)	1727.8 (560.19)	1596.9 (543.0)	1725.2 (568.7)	1863.5 (537.1)	<0.001
E-DII, Mean (SD)	0.04 (1.8)	-2.01 (1.0)	0.15 (0.5)	2.02 (0.7)	<0.001
Sex hormones, Median (Interquartile range)					
TT (ng/dl)	17.9 (12.3,25.8)	17.1 (11.4,24.1)	17.8 (12.8,23.3)	19.9 (12.7,29.0)	0.03
E2 (pg/ml)	5.8 (3.1,10.7)	5.9 (2.1,10.6)	5.4 (3.4,9.8)	7.2 (2.4,12.4)	0.29
SHBG (nmol/l)	60.2 (41.4,85.6)	60.2 (41.0,84.0)	62.0 (43.3,86.6)	61.0 (37.7,83.4)	0.77

All the above mean, standard deviation, and percentage of frequency were weighted except for the frequency. E-DII, energy-adjusted dietary inflammation index; BMI, body mass index; TT, total testosterone; E2, estradiol; SHBG, sex hormone-binding globulin.

E-DII tertile ranges: tertile 1, -5.04 to -0.69; tertile 2, -0.69 to 1.02; tertile 3, 1.02 to 4.55.

### Association Between the E-DII and Sex Hormones

**Table 2** presented the association of E-DII with sex hormones in the linear regression model. Only ln (TT) and ln (FT) showed a significant positive association of E-DII and had a significant increasing trend (*P* < 0.05 for trend) across the tertile of E-DII. After controlling for all covariates, continuous E-DII was positively associated with ln (TT), ln (FT) and ln (TT/E2) (All *P* < 0.05). No significant association of E-DII with ln (E2), and ln (FE2), ln (SHBG) was found. When the E-DII score was grouped as tertile, only a significant increasing trend of TT/E2 (*P* = 0.04 for trend) was observed, the trend observed for other sex hormones was not significant.

**Table 2:** Linear association between E-DII and sex hormones.

E-DII	TT	FT	E2	FE2	SHBG	TT/E2
	β (95%CI)	β (95%CI)	β (95%CI)	β (95%CI)	β (95%CI)	β (95%CI)
**Crude model^†^**						
Continues	**0.04**	**0.05**	0.025	0.03	-0.016	0.016
	**(0.017, 0.066)**	**(0.028-0.077)**	(-0.04, 0.09)	(-0.03, 0.09)	(-0.04, 0.007)	(-0.043, 0.078)
Tertile 1	Reference	Reference	Reference	Reference	Reference	Reference
Tertile 2	**0.074**	**0.055**	-0.04	-0.05	0.03	0.115
	**(-0.05, 0.20)**	**(-0.08, 0.19)**	(-0.24, 0.15)	(-0.25, 0.145)	(-0.08, 0.13)	(-0.11, 0.34)
Tertile 3	**0.194**	**0.21**	0.12	0.126	-0.03	0.07
	**(0.06, 0.32)**	**(0.07, 0.34)**	(-0.14, 0.38)	(-0.14, 0.39)	(-0.14, 0.08)	(-0.185, 0.33)
*P* for trend	**0.015**	**0.008**	0.34	0.28	0.68	0.59
**Adjusted model^&^**						
Continues	**0.035**	**0.026**	-0.03	-0.036	0.012	**0.065**
	**(0.007, 0.06)**	**(0.001, 0.05)**	(-0.08, 0.02)	(-0.08, 0.01)	(-0.01, 0.03)	**(0.02, 0.11)**
Tertile 1	Reference	Reference	Reference	Reference	Reference	Reference
Tertile 2	0.056	0.005	-0.14	-0.17	0.074	**0.197**
	(-0.07, 0.185)	(-0.14, 0.15)	(-0.30, 0.024)	(-0.33, -0.008)	(-0.025, 0.17)	**(0.0005, 0.39)**
Tertile 3	0.174	0.12	-0.11	-0.147	0.077	**0.284**
	(0.02, 0.325)	(0.034, 0.27)	(-0.315, 0.01)	(-0.35, 0.05)	(-0.02, 0.17)	**(0.09, 0.47)**
*P* for trend	0.07	0.15	0.28	0.15	0.23	**0.04**

E-DII, energy-adjusted dietary inflammation index; TT, total testosterone; FT, free testosterone; E2, estradiol; FE2, free estradiol; SHBG, sex hormone-binding globulin; TT/E2, the ratio of TT to E2.

E-DII tertile ranges: tertile 1, -5.04 to -0.69; tertile 2, -0.69 to 1.02; tertile 3, 1.02 to 4.55.

^†^Unadjusted.

^&^Adjusted for age, race, education, marital status, smoking, body mass index, waist circumference, type of menopause, time since menopause, parity, time of blood sampling and energy intake.

The numbers in bold letters indicate that the AIC was considered smaller. The numbers in bold letters indicate that the result was considered significant difference.

**Figure 2:**
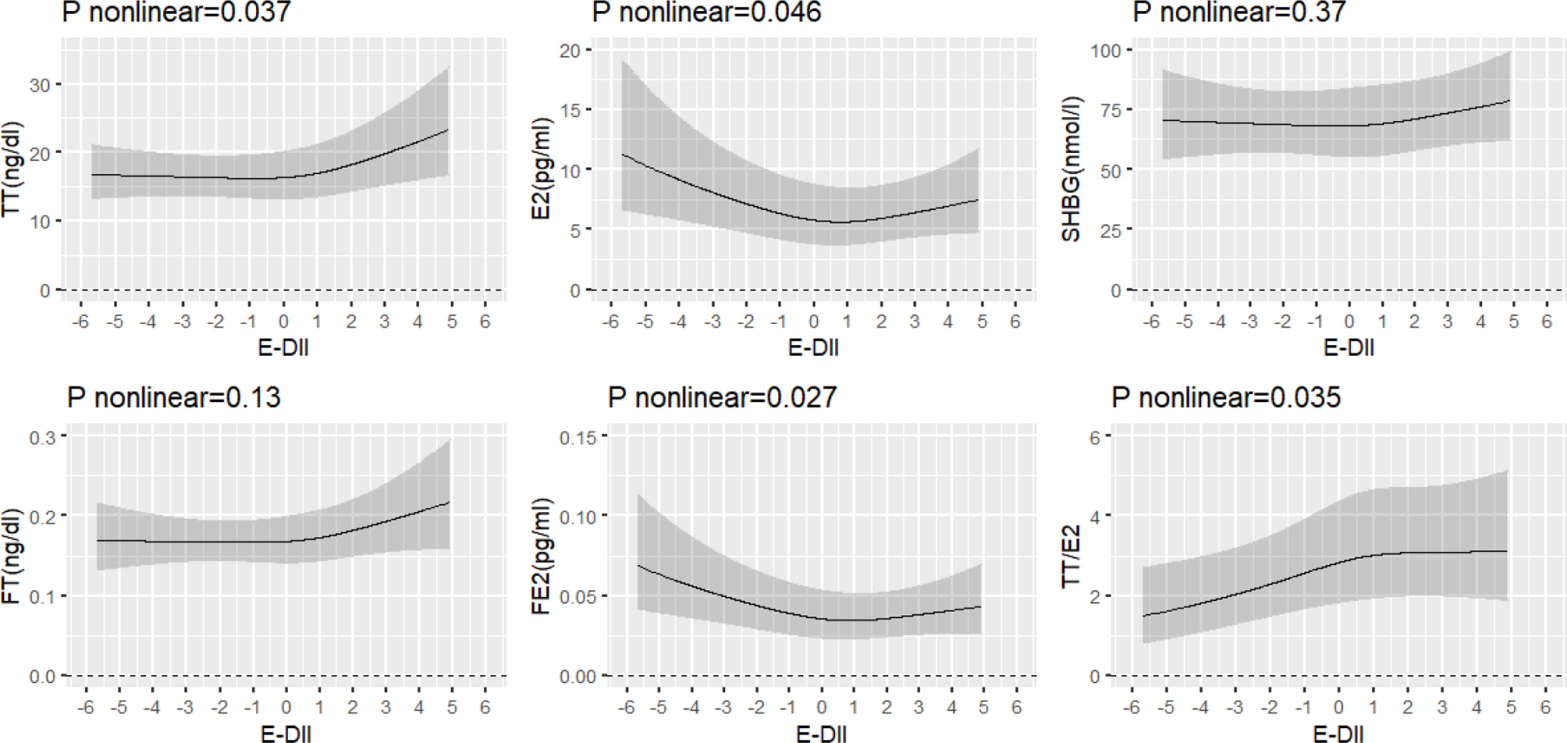
Restricted cubic spline models of association between E-DII and sex hormones. E-DII, energy-adjusted dietary inflammation index; TT, total testosterone; FT, free testosterone; E2, estradiol; FE2, free estradiol; SHBG, sex hormone-binding globulin; TT/E2, the ratio of TT to E2. All models were adjusted for age, race, education, marital status, smoking, body mass index, waist circumference, type of menopause, time since menopause, parity, time of blood sampling and energy intake.

Restricted cubic spline model was conducted to further explore the nonlinear association between E-DII and sex hormones (**Figure 2**). The restricted cubic splines suggested a consistent U-shaped association of ln (E2) (*P*-nonlinear = 0.046) and ln (FE2) (*P*-nonlinear = 0.027) with E-DII, the two inflection points were found at the E-DII score of -0.22 and 0.07, respectively. The non-linear association of ln (TT) (*P*-nonlinear = 0.037) and ln (TT/E2) (*P*-nonlinear = 0.035) with E-DII also were found. With the increase of E-DII score, ln (TT) flattened until the E-DII score was around 0.4 and increased gradually afterward, while ln (TT/E2) increased until the E-DII score was around 0.75 and then flattened out at a plateau. ln (FT) (*P*-nonlinear = 0.13) and ln (SHBG) (*P*-nonlinear = 0.37) didn’t show any nonlinear association with E-DII. For the association between E-DII and sex hormones, the restricted cubic spline model was better fit than the linear model based on AIC values (**Supplementary Table 1**).

### Subgroup Analyses

In terms of natural menopause women, the U-shaped association of E-DII with ln (E2) and ln (FE2) was still apparent and those curves of association between E-DII and other sex hormones remained very similar (**Supplementary Figures 1–6** and **Supplementary Table 2**). No significant association was observed in women with hysterectomy. Overall, the linear and nonlinear associations of E‐DII with sex hormones in natural menopause women were relatively stronger than in all postmenopausal women (**Table 3**). When stratified by BMI, the association between E-DII and most sex hormones was nonsignificant. The curve describing the nonlinear association also didn’t seem to differ in different BMI groups (**Table 4**).

**Table 3:** Association between E-DII and sex hormones stratified by type of menopause.

Type of menopause	TT	FT	E2	FE2	SHBG	TT/E2
	β (95%CI)	β (95%CI)	β (95%CI)	β (95%CI)	β (95%CI)	β (95%CI)
**Hysterectomy (n=288)**						

Continues	-0.001	0.004	0.02	0.02	-0.01	-0.02
	(-0.06, 0.06)	(-0.04, 0.05)	(-0.06, 0.1)	(-0.05, 0.1)	(-0.05, 0.03)	(-0.09, 0.05)
Tertile 1	Reference	Reference	Reference	Reference	Reference	Reference
Tertile 2	-0.024	-0.06	0.1	0.08	0.04	-0.13
	(-0.34, 0.29)	(-0.33, 0.22)	(-0.25, 0.45)	(-0.27, 0.43)	(-0.12, 0.21)	(-0.61, 0.36)
Tertile 3	-0.037	-0.03	0	0	-0.01	-0.04
	(-0.40, 0.33)	(-0.33, 0.27)	(-0.34, 0.34)	(-0.33, 0.33)	(-0.17, 0.14)	(-0.39, 0.31)
*P* for trend	0.98	0.88	0.83	0.88	0.76	0.86
**Natural menopause (n=895)**						

Continues	**0.05**	**0.03**	-0.05	-0.06	0.02	**0.10**
	**(0.01, 0.08)**	**(0.002, 0.06)**	(-0.1, 0.01)	(-0.1, 0.026)	(-0.009, 0.04)	**(0.06, 0.14)**
Tertile 1	Reference	Reference	Reference	Reference	Reference	Reference
Tertile 2	**0.08**	0.03	**-0.26**	**-0.28**	0.07	**0.33**
	**(-0.06, 0.22)**	(-0.14, 0.2)	**(-0.4, 0.11)**	**(-0.45, -0.12)**	(-0.05, 0.19)	**(0.18, 0.49)**
Tertile 3	**0.25**	0.17	**-0.16**	**-0.21**	0.11	**0.41**
	**(0.07, 0.43)**	(-0.02, 0.36)	**(0.34, 0.01)**	**(-0.39, -0.04)**	(0, 0.22)	**(0.23, 0.6)**
*P* for trend	**0.02**	0.86	**0.01**	**0.01**	0.20	**0.001**

E-DII, energy-adjusted dietary inflammation index; TT, total testosterone; FT, free testosterone; E2, estradiol; FE2, free estradiol; SHBG, sex hormone-binding globulin; TT/E2, the ratio of TT to E2.

E-DII tertile ranges: tertile 1, -5.04 to -0.69; tertile 2, -0.69 to 1.02; tertile 3, 1.02 to 4.55.

All model were adjusted for age, race, education, marital status, smoking, body mass index, waist circumference, time since menopause, parity, time of blood sampling and energy intake. The numbers in bold letters indicate that the result was considered significant difference.

**Table 4:** Association between E-DII and sex hormones stratified by BMI.

BMI	TT	FT	E2	FE2	SHBG	TT/E2
	β (95%CI)	β (95%CI)	β (95%CI)	β (95%CI)	β (95%CI)	β (95%CI)
**<25kg/m^2^ (n=276)**						
Continues	0.01	-0.008	-0.006	-0.02	0.03	0.02
	(-0.04, 0.06)	(-0.05, 0.035)	(-0.11, 0.1)	(-0.12, 0.08)	(-0.02, 0.07)	(-0.06, 0.10)
Tertile 1	Reference	Reference	Reference	Reference	Reference	Reference
Tertile 2	0.01	-0.13	-0.06	-0.15	0.18	0.08
	(-0.18, 0.20)	(-0.36, 0.11)	(-0.3, 0.17)	(-0.4, 0.1)	(0.03, 0.33)	(-0.13, 0.28)
Tertile 3	0.029	-0.11	0.02	-0.07	0.18	0.01
	(-0.18, 0.24)	(-0.36, 0.14)	(-0.24, 0.28)	(-0.35, 0.22)	(-0.01, 0.38)	(-0.20, 0.22)
*P* for trend	0.96	0.57	0.75	0.50	0.09	0.75
**25~29.9kg/m^2^ (n=347)**						
Continues	0.02	0.006	-0.05	-0.06	0.02	0.07
	(-0.01, 0.05)	(-0.03, 0.04)	(-0.12, 0.03)	(-0.14, 0.03)	(-0.03, 0.07)	(-0.01, 0.15)
Tertile 1	Reference	Reference	Reference	Reference	Reference	Reference
Tertile 2	**-0.12**	-0.16	-0.22	-0.25	0.06	0.11
	**(-0.32, 0.08)**	(-0.41, 0.09)	(-0.58, 0.13)	(-0.61, 0.11)	(-0.15, 0.26)	(-0.21, 0.42)
Tertile 3	**0.13**	0.06	0.15	0.02	0.09	0.28
	**(-0.06, 0.32)**	(-0.13, 0.25)	(-0.53, 0.22)	(-0.6, 0.2)	(-0.12, 0.3)	(-0.08, 0.64)
*P* for trend	**0.04**	0.19	0.45	0.41	0.69	0.19
**≥30kg/m^2^(n=560)**						
Continues	**0.04**	0.04	-0.03	-0.03	0.007	**0.07**
	**(0.002, 0.08)**	(-0.004, 0.08)	(-0.09, 0.03)	(-0.1, 0.03)	(-0.03, 0.04)	**(0.008, 0.14)**
Tertile 1	Reference	Reference	Reference	Reference	Reference	Reference
Tertile 2	0.20	0.18	-0.17	-0.18	0.05	0.37
	(0.01, 0.38)	(0.02, 0.33)	(-0.4, 0.06)	(-0.42, 0.07)	(-0.09, 0.19)	(0.08, 0.66)
Tertile 3	0.22	0.17	-0.12	-0.15	0.07	0.34
	(-0.01, 0.44)	(-0.04, 0.39)	(-0.33, 0.08)	(-0.37, 0.08)	(-0.1, 0.24)	(0.04, 0.64)
*P* for trend	0.14	0.12	0.32	0.33	0.7	0.07

E-DII, energy-adjusted dietary inflammation index; TT, total testosterone; FT, free testosterone; E2, estradiol; FE2, free estradiol; SHBG, sex hormone-binding globulin; TT/E2, the ratio of TT to E2.

E-DII tertile ranges: tertile 1, -5.04 to -0.69; tertile 2, -0.69 to 1.02; tertile 3, 1.02 to 4.55.

All models were adjusted for age, race, education, marital status, smoking, waist circumference, type of menopause, time since menopause, parity, time of blood sampling and energy intake. The numbers in bold letters indicate that the result was considered significant difference.

### Sensitivity Analysis

The association between E-DII and sex hormones didn’t emerge substantial change when the NHANES sampling weight was ignored (**Supplementary Tables 3, 4**). continuous E-DII appeared new association with some of sex hormones in unadjusted linear regression model. When those models were adjusted for all covariates, E-DII appeared a significant positive association with ln (SHBG), other associations changed little. In the restricted cubic spline analysis, those curves, representing the nonlinear association between E-DII and sex hormones, were generally similar (**Supplementary Figure 7**).

## Discussions

To our knowledge, this study was the first to examine the association between the E-DII and sex hormones in postmenopausal women. In this large cross-sectional study, our results found nonlinear associations of E-DII with TT, E2, FE2 and TT/E2 in postmenopausal women. These associations were more apparent in natural menopause women.

Some dietary components have been studied that could modulate sex hormones, such as whole grains, vegetables, and other foods rich in fiber (2–4). But the combined effect of various foods was more complicated. There are many possible mechanisms by which diet affects sex hormones. Our study is the first report to focus on the effects of diet-induced inflammation on sex hormones among menopause women. Our findings demonstrated that the E-DII was not significantly associated with SHBG, and a U-shaped association was observed between estrogen concentration (estradiol and free estradiol) and E-DII. We observed that participants who had neither anti-inflammatory nor pro-inflammatory diet were likely to have lower estrogen concentration. Previous studies have proved that the increases in inflammatory cytokines, including IL-6 and TNFα, have been indicated that could contribute to an increase in androgens and stimulate aromatase activity so as to promote estrogen synthesis in postmenopausal women (31, 32). While participants were on a pro-inflammatory diet, with E-DII increased, the concentrations of estrogen and androgen followed an overall downward trend. These are consistent with the proposed mechanism of peripheral estrogen synthesis regulation in previous studies. Furthermore, The Alberta Physical Activity and Breast Cancer Prevention Trial found that TT, E2, and FE2 were significantly positively associated with CRP in postmenopausal women without hormone therapy (33). Some studies also have reported that TT and E2 were positively and independently associated with a proinflammatory state in postmenopausal women (2, 23, 34). Similarly, anti-inflammatory diet was associated with low estrogen concentration in postmenopausal women (1, 4, 24). This is the contrary to our results. Whereas there is also evidence that as estrogen synthesis declines, the expression and secretion of inflammatory cytokines spontaneously increases, such as IL-6, and TNF-α (35). There may be multiple interactions between inflammatory cytokines and sex hormones. It has traditionally been considered that the biological mechanisms underlying sex hormones synthesis regulation are complex. The effects of E-DII on sex hormones may be a multifactorial process that involves the interaction of multiple mechanisms. More research is needed to further understand these complex nonlinear associations and attempt to explain the underlying mechanisms.

The curve showing association between sex hormones and E-DII in women with natural menopause was observed to be substantially different from that in women with hysterectomy, suggesting possible effect modification by type of menopause, although the interaction terms were not significant when tested and adjusted for covariates. Our study indicated that the association between E-DII and sex hormones was significant only in natural postmenopausal women. The presence of ovaries may influence sex hormones and inflammatory cytokines. Natural postmenopausal women had an increase in inflammatory cytokines, as well as those without their ovaries, but women went through hysterectomy without bilateral oophorectomy did not (36). In addition, considering that adipose tissue becomes the main source of estradiol after menopause, we conducted stratification analyses of BMI (37). In terms of our findings, obese women were observed to had higher androgen concentration than normal-weight women, but BMI has little or no effect on the association between E-DII and sex hormones. Visceral adipose tissue-induced inflammation appeared to play a major role on regulation of androgen concentrations (38), but the role of adipose tissue on the association between E-DII and sex hormones in menopause women remains to be determined. More future large-scale studies are needed to study this tissue.

This study uses data from a large, representative sample of the general United States population and has several strengths, including collecting two 24-hour dietary recall (working days and rest days) surveys to assess day-to-day dietary intake and the selection of E-DII as an indication. E-DII can be used across various dietary cultures and is superior to other dietary indicators and patterns. This study also had several limitations. One such limitation is the cross-sectional nature of NHANES severely limits causal inference. Another potential limitation is that both menopausal state and 24-hour dietary intake are self-reported, and the questionnaires may be subject to reporting bias. In addition, only 27 of 45 food parameters were available for E-DII calculation, the calculated DII might underestimate the participant’s true DII, and as a result, the observed association between DII and sex hormones might be underestimated. Although previous studies have been shown that the predictive ability of E-DII calculated from 27 foods parameters has no change in assessing inflammation compared with the full list (39, 40). Further, the effect of diet on sex hormones is a multi-mechanism process (41). In the analysis of the association between sex hormones and E-DII, the effect of other potential aspects of diet on sex hormones cannot be ruled out. Finally, lack of data on involvement in steroidogenesis, gonadotropins and gonadotropin-releasing hormones has hampered further analysis of the underlying biological mechanisms.

Altogether, our study suggested that the relationship of androgen and estrogen with E-DII was nonlinear in postmenopausal women, the U-shaped nonlinear effect of the E-DII on estrogen was observed. Our findings may provide a new perspective regarding the roles of pro- or anti-inflammation diet in the sex hormones related metabolic dysfunctions. However, in the lack of direct evidence, further studies are needed to be confirmed and understand the biological mechanisms.

## Data Availability Statement

The original contributions presented in the study are included in the article/**Supplementary Material**. Further inquiries can be directed to the corresponding author.

## Ethics Statement

The NHANES obtained approval from the National Center for Health Statistics Research Ethics Review Board and participants provided written consent and have been performed in accordance with the ethical standards as laid down in the 1964 Declaration of Helsinki and its later amendments or comparable ethical standards. The patients/participants provided their written informed consent to participate in this study.

## Author Contributions

W-YC, Y-PF, WZ, and MZ contributed to the study’s conception and design. W-YC, Y-PF, and WZ designed the study. W-YC and Y-PF collected and analyzed the data. W-YC and MZ drafted the manuscript. W-YC and Y-PF finalized the manuscript. All authors contributed to revise the article critically for important intellectual content and approve the version to be published.

## Conflict of Interest

The authors declare that the research was conducted in the absence of any commercial or financial relationships that could be construed as a potential conflict of interest.

## Publisher’s Note

All claims expressed in this article are solely those of the authors and do not necessarily represent those of their affiliated organizations, or those of the publisher, the editors and the reviewers. Any product that may be evaluated in this article, or claim that may be made by its manufacturer, is not guaranteed or endorsed by the publisher.
