# Supplementary material for: The Association Between Dietary Inflammatory Index and Sex Hormones Among Postmenopausal Women in the US
Source: Front Endocrinol (Lausanne). 2021 Dec 21;12:771565. doi: 10.3389/fendo.2021.771565 (PMC8728879; doi:10.3389/fendo.2021.771565)
Supplement: Supplementary file 1 [file DataSheet_1.docx]

Supplementary Material

# Supplementary Tables

S Table 1. Comparison of AIC values for the linear and Restricted cubic splines models of association between E-DII and sex hormones

| Sex hormones | AIC | |
| --- | --- | --- |
|  | Linear model | Restricted cubic splines model |
| TT | 408.4 | **407.2** |
| FT | 435.0 | **433.7** |
| E2 | 841.0 | **832.5** |
| FE2 | 865.7 | **859.5** |
| SHBG | **229.2** | 229.6 |
| TT/E2 | 735.4 | **733.3** |

AIC, Akaike's Information Criterion; E-DII, energy-adjusted dietary inflammation index; TT, total testosterone; FT, free testosterone; E2, estradiol; FE2, free estradiol; SHBG, sex hormone-binding globulin; TT/E2, the ratio of TT to E2.

All models were adjusted for age, race, education, marital status, smoking, body mass index, waist circumference, type of menopause, time since menopause, parity, time of blood sampling and energy intake. The numbers in bold letters indicate that the AIC was considered smaller.

S-Table 2. Comparison of AIC values for the linear and nonlinear models in subgroup analyses of association between E-DII and sex hormones

|  | TT | FT | E2 | FE2 | SHBG | TT/E2 |
| --- | --- | --- | --- | --- | --- | --- |
| **Hysterectomy****†** |  |  |  |  |  |  |
| Linear | 650.3 | 640.3 | **986.4** | 979.9 | 473.9 | **926.6** |
| Restricted cubic splines | **649.4** | **635.9** | 987.5 | **980.5** | 473.9 | 928.6 |
| **Natural menopause†** |  |  |  |  |  |  |
| Linear | 2087 | **2156** | 2465 | 2523 | 1583 | 2387 |
| Restricted cubic splines | **2086** | 2158 | **2451** | **2515** | **1578** | **2381** |
| **BMI<25kg/m^2^&** |  |  |  |  |  |  |
| Linear | **591.4** | **633.3** | 845.6 | 859 | **422** | 794.1 |
| Restricted cubic splines | 593.3 | 635.2 | **840.9** | **854.6** | 424 | **789** |
| **BMI**  **25~29.9kg/m^2^&** |  |  |  |  |  |  |
| Linear | 802.5 | 805.8 | 1033 | 1033 | 657.4 | **960.2** |
| Restricted cubic splines | **798.5** | **805.5** | **1028** | **1030** | **657.3** | 961.3 |
| **BMI≥30kg/m^2^&** |  |  |  |  |  |  |
| Linear | **1341** | **1317** | **1680** | **1697** | **965.8** | **1617** |
| Restricted cubic splines | 1342 | 1318 | 1681 | 1698 | 966.9 | 1619 |

AIC, Akaike's Information Criterion; E-DII, energy-adjusted dietary inflammation index; BMI, body mass index; TT, total testosterone; FT, free testosterone; E2, estradiol; FE2, free estradiol; SHBG, sex hormone-binding globulin; TT/E2, the ratio of TT to E2.

†, adjusted for age, race, education, marital status, body mass index, smoking, waist circumference, time since menopause, parity, time of blood sampling and energy intake.

&**,** adjusted for age, race, education, marital status, smoking, waist circumference, type of menopause, time since menopause, parity, time of blood sampling and energy intake. The numbers in bold letters indicate that AIC was considered smaller.

S-Table 3. Linear association between E-DII and sex hormones.

| E-DII | TT  β (95%CI) | FT  β (95%CI) | E2  β (95%CI) | FE2  β (95%CI) | SHBG  β (95%CI) | TT/E2  β (95%CI) |
| --- | --- | --- | --- | --- | --- | --- |
| **Crude model†** |  |  |  |  |  |  |
| Continues | **0.03**  **(0.013-0.05)** | **0.03**  **(0.01-0.05)** | **0.08**  **(0.05, 0.11)** | **0.08**  **(0.05, 0.11)** | -0.0006  (-0.02, 0.016) | **-0.046**  **(-0.07, -0.02)** |
| *P* | **0.001** | **0.002** | **<0.001** | **<0.001** | 0.94 | **0.002** |
| **Adjusted model&** |  |  |  |  |  |  |
| Continues | **0.02**  **(0.002-0.04)** | 0.016  (-0.01, 0.03) | 0.02  (-0.008, 0.05) | 0.02  (-0.08, 0.01) | **0.016**  **(0.002, 0.03)** | -0.004  (-0.03, 0.02) |
| *P* | **0.03** | 0.27 | 0.06 | 0.17 | **0.04** | 0.74 |

E-DII, energy-adjusted dietary inflammation index; TT, total testosterone; FT, free testosterone; E2, estradiol; FE2, free estradiol; SHBG, sex hormone-binding globulin; TT/E2, the ratio of TT to E2.

**†,** unadjusted.

&**,** adjusted for age, race, education, marital status, smoking, body mass index, waist circumference, type of menopause, time since menopause, parity, time of blood sampling and energy intake.

The numbers in bold letters indicate that the AIC was considered smaller. The numbers in bold letters indicate that the result was considered significant difference.

S Table 4. Sensitivity analysis (AIC values for the linear and Restricted cubic splines models of association between E-DII and sex hormones)

| Sex hormones | AIC | |
| --- | --- | --- |
|  | Linear model | Restricted cubic splines model |
| TT | **2148.3** | 2149.1 |
| FT | **2221.0** | 2221.8 |
| E2 | 2922.0 | **2919.9** |
| FE2 | 2957.1 | **2955.5** |
| SHBG | **1549.0** | 1550.8 |
| TT/E2 | **2695.0** | 2695.1 |

AIC, Akaike's Information Criterion; E-DII, energy-adjusted dietary inflammation index; TT, total testosterone; FT, free testosterone; E2, estradiol; FE2, free estradiol; SHBG, sex hormone-binding globulin; TT/E2, the ratio of TT to E2.

All models were unweighted and adjusted for age, race, education, marital status, smoking, body mass index, waist circumference, type of menopause, time since menopause, parity, time of blood sampling and energy intake. The numbers in bold letters indicate that the AIC was considered smaller.

# Supplementary Figures


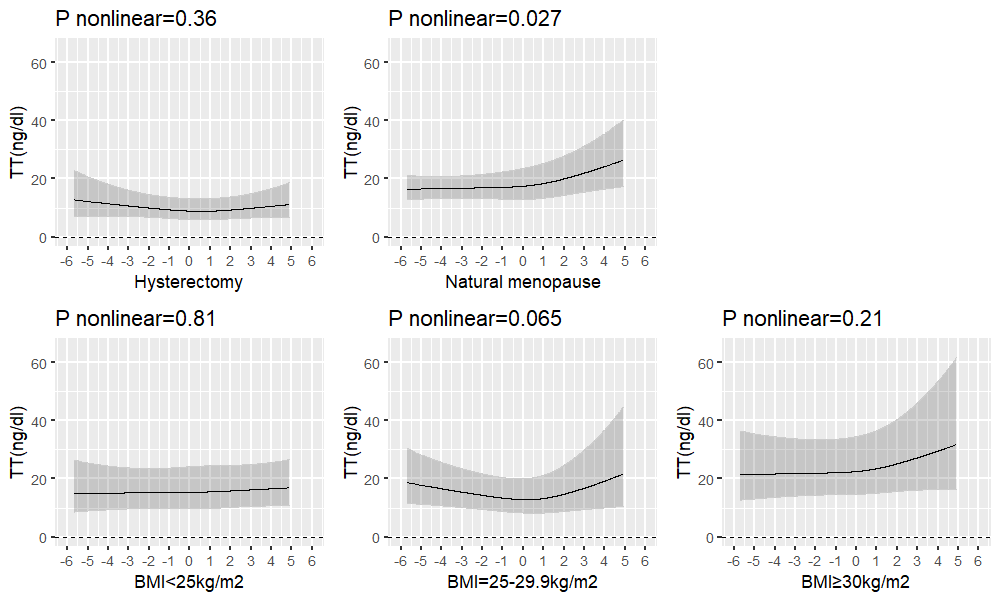


S-Figure 1. Restricted cubic splines models of association between E-DII and TT stratified by type of menopause and BMI

E-DII, energy-adjusted dietary inflammation index; TT, total testosterone. Models stratified by type of menopause were adjusted for all covariates except for type of menopause. Models stratified by body mass index were adjusted for all covariates except for body mass index.


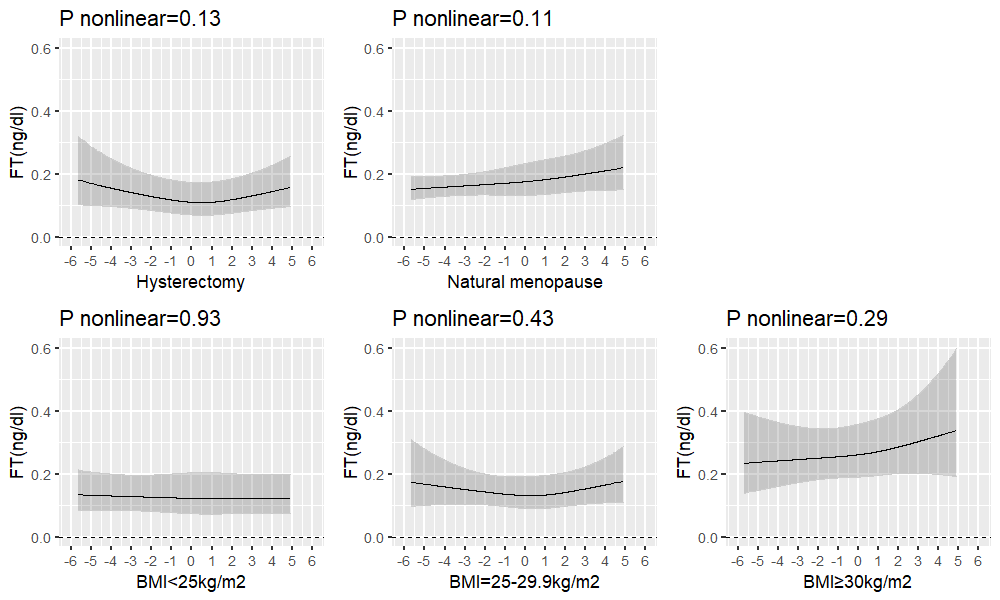


S-Figure 2. Restricted cubic splines models of association between E-DII and FT stratified by type of menopause and BMI

E-DII, energy-adjusted dietary inflammation index; FT, free testosterone. Models stratified by type of menopause were adjusted for all covariates except for type of menopause. Models stratified by body mass index were adjusted for all covariates except for body mass index.


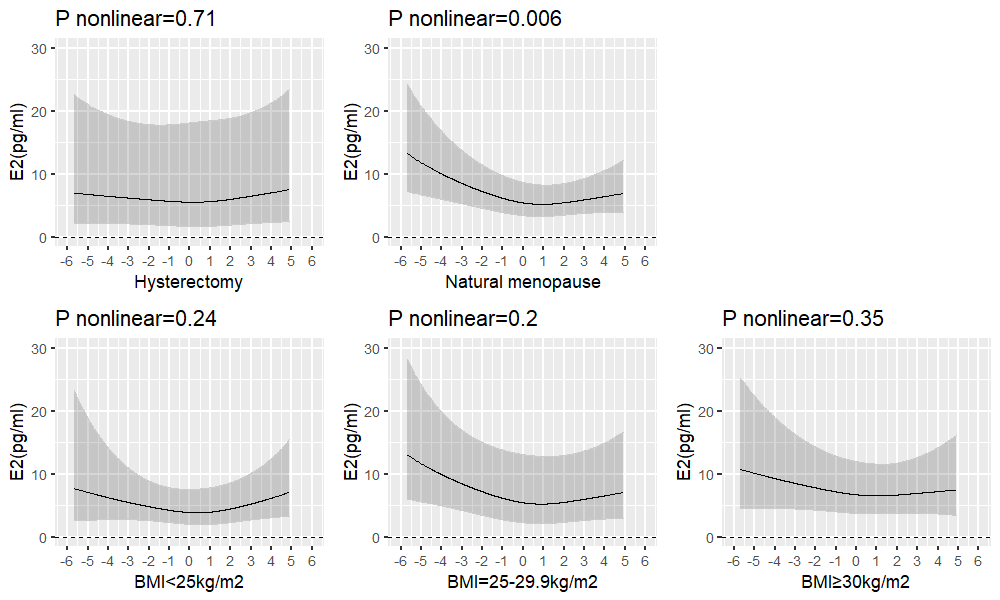


S-Figure 3. Restricted cubic splines models of association between E-DII and E2 stratified by type of menopause and BMI

E-DII, energy-adjusted dietary inflammation index; E2, estradio. Models stratified by type of menopause were adjusted for all covariates except for type of menopause. Models stratified by body mass index were adjusted for all covariates except for body mass index.


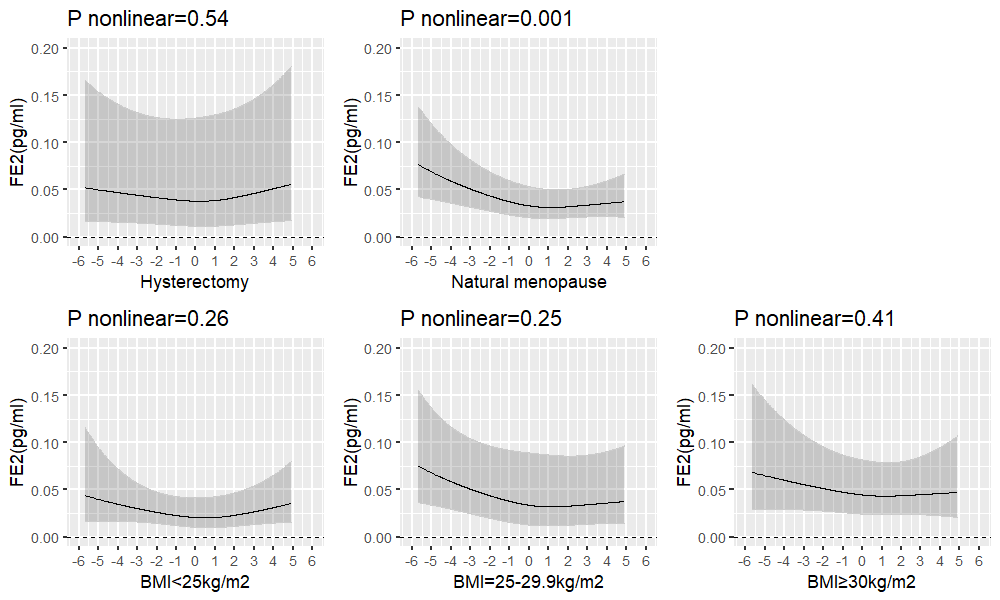


S-Figure 4. Restricted cubic splines models of association between E-DII and FE2 stratified by type of menopause and BMI

E-DII, energy-adjusted dietary inflammation index; FE2, free estradiol. Models stratified by type of menopause were adjusted for all covariates except for type of menopause. Models stratified by body mass index were adjusted for all covariates except for body mass index.


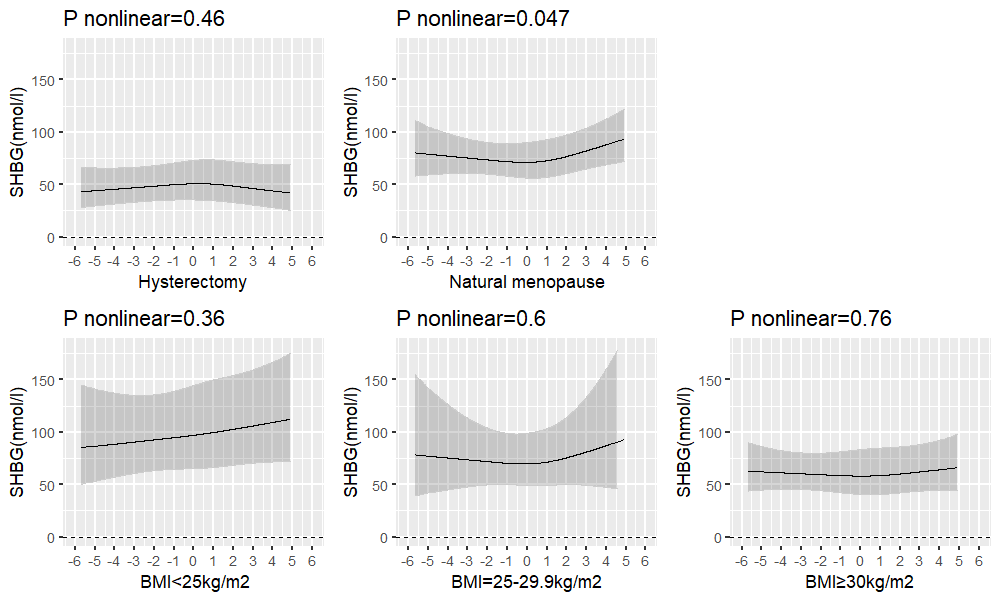


S-Figure 5. Restricted cubic splines models of association between E-DII and SHBG stratified by type of menopause and BMI

E-DII, energy-adjusted dietary inflammation index; SHBG, sex hormone-binding globulin. Models stratified by type of menopause were adjusted for all covariates except for type of menopause. Models stratified by body mass index were adjusted for all covariates except for body mass index.


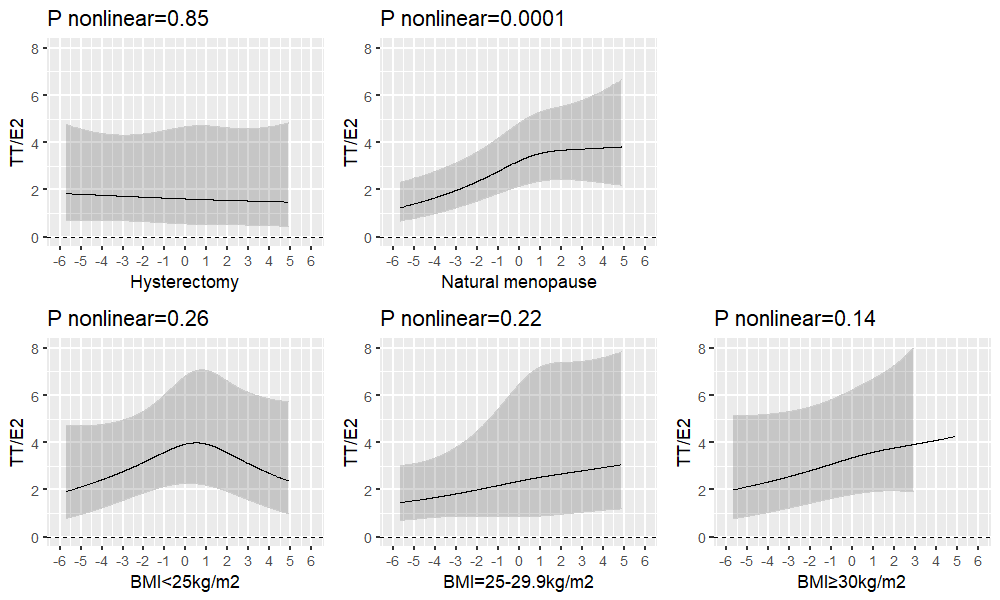


S-Figure 6. Restricted cubic splines models of association between E-DII and TT/E2 stratified by type of menopause and BMI

E-DII, energy-adjusted dietary inflammation index; TT/E2, the ratio of TT to E2. Models stratified by type of menopause were adjusted for all covariates except for type of menopause. Models stratified by body mass index were adjusted for all covariates except for body mass index.


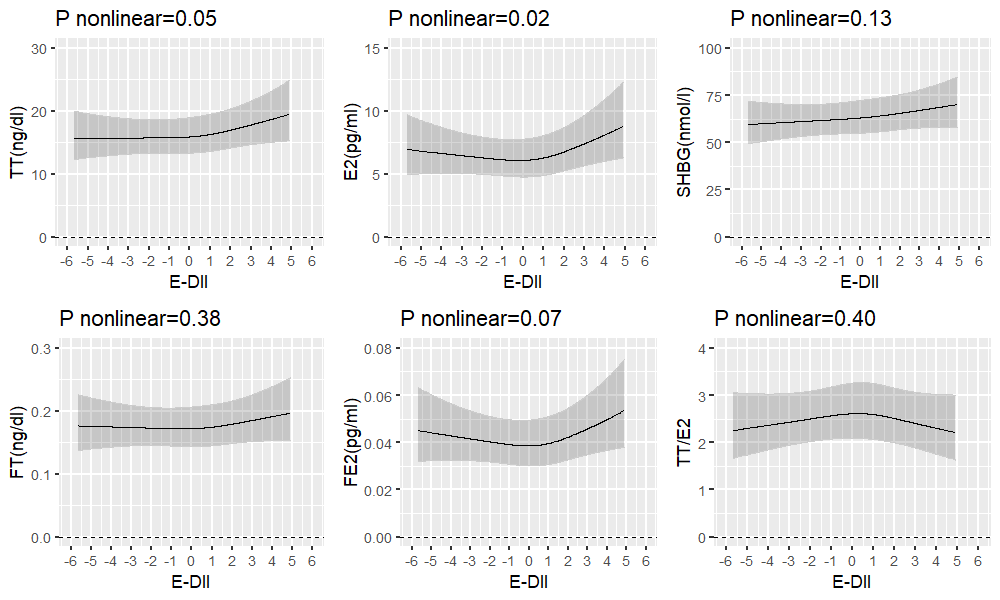


S-Figure 7. Sensitivity analysis (Restricted cubic splines models of association between E-DII and sex hormones)

E-DII, energy-adjusted dietary inflammation index; TT, total testosterone; FT, free testosterone; E2, estradiol; FE2, free estradiol; SHBG, sex hormone-binding globulin; TT/E2, the ratio of TT to E2.

All models were unweighted and adjusted for age, race, education, marital status, smoking, body mass index, waist circumference, type of menopause, time since menopause, parity, time of blood sampling and energy intake.
